# Be Healthe for Your Heart: Protocol for a Pilot Randomized Controlled Trial Evaluating a Web-Based Behavioral Intervention to Improve the Cardiovascular Health of Women With a History of Preeclampsia

**DOI:** 10.3389/fcvm.2019.00144

**Published:** 2019-09-26

**Authors:** Rachael Taylor, Vanessa A. Shrewsbury, Lisa Vincze, Linda Campbell, Robin Callister, Felicity Park, Tracy Schumacher, Clare Collins, Melinda Hutchesson

**Affiliations:** ^1^School of Health Sciences, Priority Research Centre for Physical Activity and Nutrition, Faculty of Health and Medicine, The University of Newcastle, Newcastle, NSW, Australia; ^2^School of Allied Health Sciences, Griffith University, Gold Coast, QLD, Australia; ^3^School of Psychology, Faculty of Science, The University of Newcastle, Newcastle, NSW, Australia; ^4^School of Biomedical Sciences and Pharmacy, Priority Research Centre for Physical Activity and Nutrition, Faculty of Health and Medicine, The University of Newcastle, Newcastle, NSW, Australia; ^5^Department of Maternal Fetal Medicine, John Hunter Hospital, Newcastle, NSW, Australia; ^6^Department of Rural Health, Faculty of Health and Medicine, University of Newcastle, Tamworth, NSW, Australia

**Keywords:** cardiovascular disease, preeclampsia, post-partum, health behavior, women, prevention

## Abstract

**Background:** Women with a history of preeclampsia are at greater risk of cardiovascular disease (CVD) related morbidity. Despite this knowledge, there is a lack of interventions available for women with a history of preeclampsia for the prevention of CVD. The aim of this pilot randomized controlled trial (RCT) is to determine the acceptability and preliminary efficacy of a web-based behavioral intervention targeted to women with a history of preeclampsia (Be Healthe for your Heart).

**Method:** Australian women aged 18–45 years, with a recent history (≤4 years post diagnosis) of preeclampsia will be recruited for a 3-months, 2-arm parallel group pilot RCT. Participants will be randomized into 2 study arms: (1) Be Healthe for your Heart or; (2) Control, with assessments conducted at baseline, and after 3-months. Be Healthe for your Heart is an intervention delivered online via the program website, with weekly emails to support changes in modifiable CVD risk factors (excess body weight, physical inactivity, poor diet, and stress), using behavior change techniques (e.g., self-monitoring, goal setting). Intervention acceptability (satisfaction, usability, appropriateness, and usage) and impact on absolute full CVD 30-years risk score, CVD risk markers, and modifiable risk factors will be assessed.

**Discussion:** No studies to date have evaluated acceptability and preliminary efficacy of a web-based intervention for the prevention of CVD in this high-risk population with preeclampsia. This pilot trial will inform development of a fully powered RCT if acceptability and preliminary efficacy are demonstrated.

## Introduction

Preeclampsia is a complex medical disorder in pregnancy, resulting in hypertension and multi-organ dysfunction (1). Globally, preeclampsia affects ~2–8% of pregnancies per year (2), including 3% of pregnancies in Australia (3). There is increasing evidence that preeclampsia influences women's long-term cardiovascular health. A recent meta-analysis of 22 studies including more than 6.4 million women, found that preeclampsia was significantly associated with future risk of heart failure [Relative Risk (RR): 4.19, 95% Confidence Interval (CI): 2.09–8.38], coronary heart disease (RR: 2.50, 95% CI: 1.43–4.37), cardiovascular disease (CVD) mortality (RR: 2.21, 95% CI 1.83–8.26), and stroke (RR: 1.81, 95% CI: 1.29–2.55) (4). Other systematic reviews support this evidence of increased risk of CVD among women with a history of preeclampsia (5–7).

Current clinical guidelines acknowledge that preeclampsia is a primary CVD risk factor. For example, the 2018 Multi-society Guideline on the Management of Blood Cholesterol (8), list a history of preeclampsia as a key risk factor. The Society of Obstetric Medicine of Australia and New Zealand (SOMANZ) Guideline for the Management of Hypertensive Disorders of Pregnancy (9), also acknowledges hypertension and CVD as potential long-term consequences of preeclampsia. The guidelines recommend counseling women post pregnancy regarding key modifiable risk factors for CVD (e.g., excess body weight, physical inactivity, poor diet), along with annual blood pressure monitoring, and a minimum of 5-yearly assessments of serum lipids and blood glucose.

Many women with a history of preeclampsia remain unaware that preeclampsia influences their lifetime cardiovascular health, and few are receiving the recommended monitoring and advice. An Australian survey of 127 women who had been diagnosed with preeclampsia within the last 2 years found 34.1% were unaware of their increased risk of CVD (10). While 94.5% of the entire sample reported recent monitoring of their blood pressure, < half (40.5%) reported monitoring of their serum lipids and/or blood glucose (40.9%), and <25% had received advice on modifiable CVD risk factors (10).

To date, limited research has evaluated the efficacy of different intervention approaches addressing cardiovascular health post pregnancy for women with a history of preeclampsia. To the authors' knowledge there has been one recent trial published from the United States (11) and another currently underway in Australia (12). Therefore, the aim of this pilot randomized controlled trial (RCT) is to determine the acceptability and preliminary efficacy of a web-based lifestyle behavioral intervention targeted to women with a recent history of preeclampsia (Be Healthe for your Heart). The study will:

1. Evaluate intervention acceptability (satisfaction, usability, appropriateness, and usage).
2. Estimate intervention impact on absolute full CVD 30-years risk score, CVD risk markers (body fat percentage, body mass index (BMI), waist circumference, blood pressure and blood lipids, and glucose), health behavior risk factors (dietary intake, physical activity, and stress) and general health and well-being, from pre to post-intervention compared with the control group.

## Materials and Methods

### Study Design and Setting

Be Healthe for your Heart is a 3-months, 2-arm parallel group pilot RCT which is being undertaken at The University of Newcastle, New South Wales (NSW), Australia. Participants will be randomized into two study arms: (1) Be Healthe for your Heart or (2) Control, with assessments conducted at baseline and after 3-months. The study was prospectively registered with the Australian New Zealand Clinical Trials Registry (ANZCTR): 12618001528246. The template for intervention description and replication (TIDieR) checklist and guide (13) and the CONsolidated Standards of Reporting Trials (CONSORT) extension for randomized pilot and feasibility trials 2010 checklist (14) were applied for the reporting of this study. The funding bodies have no role in the design, conduct or reporting of the trial.

The pilot study received ethics approval from the Hunter New England Human Research Ethics Committee (18/09/19/4.09) and is registered with The University of Newcastle Human Research Ethics Committee. The trial will be undertaken in compliance with the Declaration of Helsinki (15). Prior to study enrolment, all participants will provide electronic or written informed consent for the 3-months study duration. Participants will be informed that they may withdraw from the study at any time without having to give a reason.

### Participants: Eligibility, Recruitment, and Eligibility

Women aged 18–45 years with a recent history (within 4 years of diagnosis) of preeclampsia will be targeted for recruitment. Inclusion and exclusion criteria are summarized in Table 1. Notably, women were excluded if they required ongoing medical follow-up after their 6-weeks postpartum check-up or have been diagnosed with type 1 or type 2 diabetes, as health conditions and their associated treatment may impact on the preliminary efficacy outcomes.

**Table 1:** Participant inclusion and exclusion criteria.

**Inclusion criteria**	**Exclusion criteria**
History of preeclampsia (within 4 years of diagnosis)	Currently or recently pregnant (<3 months post-partum)
Aged 18–45 years	Planning to become pregnant within the next 3-months
Internet access and email address	Non-English speaking
Able to attend assessments at The University of Newcastle Callaghan campus	Type 1 or 2 diabetes mellitus
Interested in all or some of the topics below: a) Improving eating habits b) Improving physical activity levels c) Managing their weight d) Managing their stress	Currently participating in another lifestyle behavior intervention
Self-reported completion of post-partum check-up at 6 weeks with no further follow-up required	Unable to provide the contact details of a General Practitioner to share their physical measurements and blood test results with, for further follow-up if required.

Within the study setting (Hunter region, NSW, Australia) there is currently no routine system for follow-up of women with a history of preeclampsia to provide cardiovascular risk assessment or preventative health services. Therefore, the study will investigate a variety of strategies to reach and recruit the target population for the intervention. All potential participants screened for eligibility will be asked how they found out about the study to evaluate the effectiveness of different recruitment strategies. Participants will be recruited using the following strategies:

1. Emailing invitations to women who previously (April 2018) completed a “Preeclampsia Survey” (10) from The University of Newcastle and agreed to be contacted about the study.
2. Advertising on the Australian Action for Preeclampsia social media accounts and online newsletter.
3. Mailing invitations to all women who were treated at John Hunter Hospital, NSW, Australia for preeclampsia within the last 4-years.
4. Providing study details to General Practitioners within the Hunter New England and Central Coast regions via the Primary Health Network newsletter and a researcher visiting medical centers in close proximity to the University to encourage their participation. General Practitioners will be asked to provide information about the study to appropriate women who meet the inclusion criteria during standard consultations as well as advertise the study in waiting rooms using the provided flyers and posters.
5. Services that have contact with women within 4 years of birth such as childcare centers, playgroups, child recreation activities, and community centers will be asked via email or phone to advertise the study using their social media accounts or by displaying posters and/or flyers on their premises.

All recruitment materials including social media posts, posters and flyers direct women to the online participant information statement describing the study and an online survey to assess eligibility for participation in the study. Participants deemed eligible will be emailed or mailed a consent form. Ineligible participants will be contacted via email or phone to inform them of the outcome.

### Randomization, Blinding, and Sample Size

The randomization sequence will be generated by an independent statistician, using a random number function in Microsoft Excel. Concealed envelopes will be distributed to the participants after baseline measurements for randomization to each study condition (1:1). A randomized block design, with a block size of 6, will be used to ensure the conditions are balanced. Randomization will be stratified by time since last pregnancy complicated by preeclampsia at time of enrolment (3 months to <1 year, ≥1 to <2 years, ≥2 to 4 years). Participant blinding will not be possible because they will be aware of the trial's conditions and the differences will be apparent. Researchers involved in the collection of physical measurements will be blinded to participant group allocation until completion of the 3-months follow-up appointment. The researchers will advise participants at the 3-months follow-up appointment that they cannot discuss their group allocation. A powered sample is not required for a pilot study, so a maximum of 90 participants (45/per group) will be recruited, as this is feasible within the funding timeline and budget.

### Intervention

#### Intervention Development

The intervention delivery mode, duration and content were informed by formative research in Australian women with a history of preeclampsia, conducted via an online survey (10). Of the 100 survey respondents who provided feedback on intervention development, 96% indicated they were interested in participating in a lifestyle behavior intervention for women with a history of preeclampsia. Of the 96 who were interested in participating, the most preferred mode of delivery for the program was web-based (69.8%), with far fewer wanting in-person (18.8%), or telephone (1%) delivery. The most popular web-based delivery modes were via email (73.1%) and website (50.5%). Participant preferences for web-based delivery may be due to difficulties associated with attending in-person appointments during the postpartum period due to lack of time and childcare. This is consistent with previous research that found women with pregnancy complications, such as preeclampsia, who were referred to an in-person maternal health clinic in Canada for postpartum cardiovascular risk counseling had low attendance, with 54% failing to attend their appointment (16). Participants of the online survey (10) were also asked how much time they would be willing to commit to take part in the lifestyle behavior intervention, including both the number of weeks, and hours per week. The mean number of weeks they were willing to commit was 17.6 weeks, and mean hours per week were 5.3 hours. Participants also ranked their level of interest with proposed program topics (data not shown). Data from the research survey (10), along with existing evidence for CVD prevention (17, 18), and technology-delivered interventions (19–21) informed the intervention development. Also, members of the research team had previously developed (22) and demonstrated preliminary efficacy (23) of an eHealth weight loss intervention targeted to young women, so relevant components were adapted for use in this intervention.

#### Intervention Components and Delivery

**Figure 1:**
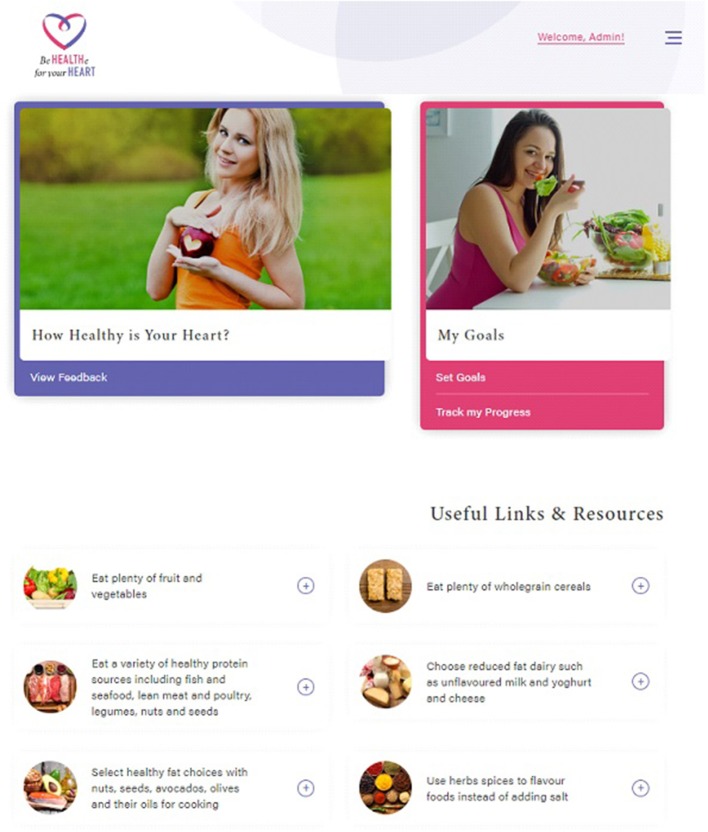
Be Healthe for your Heart website. All images are reprinted from shutterstock.com under a Standard License, with permission from Shutterstock.

Be Healthe for your Heart is a 3-months lifestyle behavior intervention delivered solely online via the program website (Figure 1) and weekly email newsletters. The program website and emails will provide participants with resources and tools related to nutrition, physical activity, stress management, and weight management consistent with the program recommendations. Program recommendations focus on improving modifiable risk factors to promote cardiovascular health and were informed by best practice guidelines (24–27). As per the Australian National Heart Foundation Heart Healthy Eating Principles, nutrition recommendations focus on eating plenty of fruit, vegetables, and wholegrain cereals, eating a variety of healthy protein sources, choosing reduced-fat dairy, selecting healthy unsaturated fat choices, and limiting salt intake through the use of herbs and spices (24). Physical activity recommendations focus on regular physical activity (most days), gradually building up to 2.5 hours of moderate intensity physical activity or 1.25 hours of vigorous intensity physical activity (or an equivalent combination of both) each week, doing muscle strengthening activities at least 2 days each week, and limiting the amount of time spent in prolonged sitting (26). Stress management recommendations focus on identifying and managing emotional stress, while weight management recommendations focus on returning to pre-pregnancy weight, and then reaching and maintaining a healthy weight (BMI 18.5–25 kg/m^2^) (27). Table 2 describes the key program components, which are aligned with 21 different behavior change techniques (28).

**Table 2:** Description of the Be Healthe for your Heart 3-months intervention.

**Program component**	**Description**	**Behavior change techniques (28)**
Website: How Healthy is your Heart?	A brief survey (37 questions) assesses the participant's eating habits, physical activity and stress levels, as well as body weight, during the first week of the program. Participants will receive automated individualized feedback on their current health behaviors compared to program recommendations. Feedback (Figure 2) will be reported for each program recommendation using a heart rating system ranging from “0 hearts = needs improvement” to “5 hearts = excellent.” Participants will also receive an overall heart score ranging from 0 to 65, with 65 indicating compliance with all program recommendations.	• Feedback on behavior • Feedback on outcomes of behavior • Problem solving • Action planning
Website: My goals	Participants will use the feedback from “How Healthy is your Heart?” to set health behavior goals during the first week of the program using the website resource “My Goals.” Participants will be able to select 1–4 goals related to eating habits, physical activity, stress and/or their weight, that are consistent with the program recommendations. Participants will be able to record their own strategies for achieving their selected goals.	• Goal setting • Action planning • Problem solving • Conserving mental resources
Website: Track my progress	Using the website resource “Track My Progress,” participants will monitor their progress toward their goals and the program recommendations. “Track My Progress” will require participants to answer questions specific to their selected goals. Based on the participant's response, automated feedback using the heart rating system, as previously described, will indicate their progress toward their goals and the program recommendations. Participants will be able to self-monitor their progress throughout the 3-months and receive feedback. Participants will be encouraged via the email newsletters to use “Track My Progress” at least once during the 3-months intervention	• Self-monitoring of behavior • Self-monitoring of outcome(s) of behavior • Feedback on behavior • Feedback on outcomes of behavior • Discrepancy between current behavior and goal • Review behavior goals • Habit formation
Website: Resources	The resources include comprehensive written information related to the program recommendations for nutrition, physical activity, stress management and weight management, consistent with best practice guidelines (25–27). The resources are designed to educate participants about improving modifiable risk factors that will assist them to achieve their goals and the program recommendations. The resources will also provide external links to supporting information, videos, recipes and phone apps. Participants will be able to access any of the resources.	• Problem solving • Instruction on how to perform a behavior • Information about antecedents • Information about health consequences • Demonstration of the behavior • Information about emotional consequences • Behavior substitution • Habit reversal • Reduce negative emotions • Conserving mental resources
Emails	Participants will receive weekly emails, which will focus on a specific program recommendation and provide relevant links to the program website tools and resources, as well as remind the participants to take the quiz “How Healthy is your Heart?”, set health goals and track their progress.	• Problem solving • Instruction on how to perform a behavior • Prompts/cues • Behavioral practice/ rehearsal • Conserving mental resources

**Figure 2:**
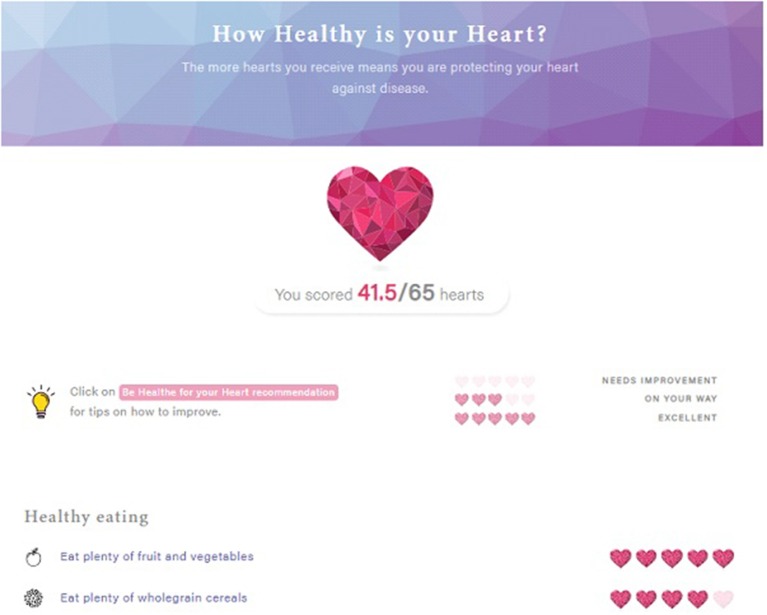
Feedback provided from “How Healthe is your Heart?”.

Participants randomized to the Be Healthe for your Heart program will be registered for the program following randomization at the baseline data collection session. They will receive an email with a link to the website (http://behealthe.newcastle.edu.au) and a username and password to log-in. Email newsletters will be automated, with the first received by participants ~2 hours after registration, and weekly thereafter. Participants will receive no instructions from researchers about how to use the website.

### Control Group

The control group will be sent an initial email with links to the National Heart Foundation of Australia website. They will receive access to the Be Healthe for your Heart program after completion of 3-months follow-up.

### Study Procedure

All eligible participants will attend measurement sessions at the University of Newcastle, Callaghan campus at baseline and 3-months. Participants will receive reminders about their scheduled measurement sessions via email and text messages. The same procedure will be followed at both time-points, participants will have their height, weight, body fat percentage, waist circumference, and blood pressure measured by researchers trained in the study protocol by the Chief Investigator. Fasting blood samples will be collected by trained phlebotomists. Participants will complete online surveys about their dietary intake, physical activity levels, general health and well-being, stress levels, breastfeeding, pregnancies, and preeclampsia history. After all measurements have been collected at baseline, participants will be randomly allocated to the intervention or control group. Participants will receive a gift voucher (AU$20 baseline and AU$40 follow-up) to reimburse them for their time and costs associated with attending study measurement sessions.

### Outcome Measures

#### Acceptability (Primary Outcome)

At 3-months, the participants in the intervention group will complete an online survey via the Qualtrics (Qualtrics, Seattle, Washington, US) platform with 40 questions related to program component usage, usability, appropriateness, satisfactions, and reasons for engagement or non-engagement. Participants will evaluate all components of the Be Healthe for your Heart website including the “How Healthy is your Heart?,” “My Goals,” “Track My Progress,” website resources and weekly emails. Survey questions will require participants to indicate their level of agreement with specific statements and describe what they liked or disliked and what could be improved for each program component. Participants will also complete survey questions related to their overall satisfaction with the program. The study will also objectively measure the use of the intervention components by recording their website logins and website page visits and number of email newsletters opened.

#### Preliminary Efficacy (Secondary Outcome)

All secondary outcomes will be measured at baseline and after 3-months to allow evaluation of change in outcomes during the intervention period to provide an indication of the immediate impact of the intervention (i.e., preliminary efficacy). As this is a pilot RCT completion of each outcome measure will also be tracked, as a measure of the feasibility of the data collection procedures. The following objective measurements will be taken:

- *Weight, BMI, waist circumference, and body fat percentage:* Each participant's height, weight, waist circumference, and body fat percentage will be measured by researchers trained in the study protocol by the Chief Investigator. Height to the nearest 0.1 cm will be measured twice using the stretch stature method on a stadiometer (Inbody BSM370; Inbody Australia, Miami, QLD, Australia). A third measurement will be obtained when the difference between repeated height measures is >0.3 cm. Weight to the nearest 0.01 kg will be measured twice in light clothing, without shoes on a digital scale. A third measurement will be obtained when the difference in repeated weight measures is >0.4 kg. The mean of the 2 measurements with the least difference will be used for analysis. Body fat percentage will be determined using bioelectrical impedance (Inbody 720; Inbody Australia, Miami, QLD, Australia). BMI will be calculated as weight (kg) divided by height (m) squared, and categorized into underweight (BMI: <18.5), healthy (BMI: ≥18.5 to <24.9), overweight (BMI: ≥24.9 to <29.9), or obese (BMI: ≥30) categories according to the cut-off points defined by the World Health Organization (WHO) (29). Waist circumference to the nearest 0.1 cm will be measured twice at the midpoint between the lower costal (10th rib) border and the top of the iliac crest, with clothing raised so that the waist is exposed, using a non-extensible steel tape (30). Waist circumference will be measured by the same researcher at both time-points to ensure that consistent measurements are collected. A third waist measurements will be taken when the difference between repeated waist measurements is >0.5 cm. The mean of the 2 measurements with the least difference will be used for analysis.
- *Systolic and Diastolic Blood pressure:* Each participant will have 3 blood pressure measurements taken using the automatic sphygmomanometer (Inbody BPBIO320, Inbody Australia, Miami, QLD, Australia) which has been validated against the European Society of Hypertension International Protocol for clinical use in adults (31). Measurement procedures will be consistent with the National Heart Foundation's Guidelines for the diagnosis and management of hypertension in adults (32). Specifically, participants will be seated for 5 min before their first blood pressure measurement, then for 2 min between the remaining measurements. When there is a difference of more than 10 mmHg between any of the systolic or any of the diastolic values, a fourth measurement will be taken. The mean of the 2 measurements with the least difference will be used for analysis. Blood pressure readings displayed on the screen will not be visible to the participants during the measurement session.
- *Cardiovascular blood tests:* Each participant will have 4 mL blood samples collected by trained phlebotomists and assayed by a NSW Health Pathology, which is accredited by the National Association of Testing Authorities. Blood samples will be collected after an overnight (8–12 hours) fast and assayed for total cholesterol (mmol/L), high-density lipoprotein cholesterol (HDL-C) (mmol/L), low-density lipoprotein cholesterol (LDL-C) (mmol/L), triglycerides (mmol/L), glucose (mmol/L), and insulin (mIU/L).
- *Overall cardiovascular health score:* Each participant's risk of CVD will be derived using the *Framingham CVD 30-years risk score* (33) which is derived using age (years), sex, total, and HDL-C (mg/dL), current smoking status (obtained from study surveys), systolic blood pressure (mmHg), use of antihypertensive treatment (obtained from study surveys), and whether they have been diagnosed with diabetes. Absolute full CVD risk (includes hard CVD or coronary insufficiency, angina pectoris, transient ischemic attack, intermittent claudication, or congestive heart failure) over 30 years will be classified as low risk (<10% or 1–2 points), intermediate risk (10–20% or 3–6 points), or high risk (>20% or 7 or more points). The *Framingham CVD 30-years risk score* has demonstrated an acceptable level of accuracy for predicting CVD risk in a cohort of adults (*n* = 4506) aged 20–59 years at baseline, based on cross-validated discrimination c = 0.803 and calibration chi-square = 4.25 (*p* = 0.894) (33).

At baseline and 3-months participants will be asked to complete online surveys administered via Qualtrics (Qualtrics, Seattle, Washington, US) to evaluate the following outcomes:

- *Physical activity duration and intensity* will be assessed using the International Physical Activity Questionnaire (IPAQ) (short-form) which has acceptable accuracy and reliability for the measurement of physical activity in adults aged 18–65 years across 12 countries (34). The IPAQ will require participants to recall the amount of time they spent in moderate activity, vigorous activity, walking and sitting in the past 7 days, and reported using metabolic equivalent of task (MET-minutes) per week. Based on the participant's responses to these questions, their level of physical activity over the previous 7 days will be categorized as either high, medium or low according to the IPAQ scoring protocol (35). Participants will also be asked additional questions about their amount (minutes per session) and frequency (times per week) of participation in resistance-based physical activity.
- *Sitting time* will be assessed using The Domain-Specific Sitting Questionnaire (adapted version) which has been validated in Australian adults (36, 37). This questionnaire includes 5-items that asks participants to report the number of hours and minutes spent sitting on each day (including weekdays and weekends) in the following 5 domains: traveling to/from work, at work, using a computer at home, watching television, and during leisure time (excluding watching television). Total daily sitting times on weekdays and non-weekdays will be calculated for each participant by summing reported sitting times across the domains.
- *Dietary intake* will be assessed using The Australian Eating Survey (AES). The AES is a validated measure of usual dietary intake in Australian adults, compared to 3-days weighed food records (38). The AES is a 120-item semi-quantitative Food Frequency Questionnaire (FFQ) with 15 supplementary questions related to age, vitamin and mineral supplement/s use and food behaviors. This study will use the CVD version of the AES which contains an additional 66 supplementary questions specific to foods and nutrients related to CVD health and has been shown to be more accurate for estimating long-chain polyunsaturated fatty acid intakes in hyperlipidaemic adults than the standard AES (39). Participants will be required to report their consumption of each food or food type, with frequency options which vary depending on the item and range from “Never” to “4 or more times per day” and for some beverages up to “7 or more glasses per day.” Each participant's intake of 53 macro-and micro-nutrients will be calculated using the AUStralian Food and NUTrient database (AUSNUT) 2011-13 (Food nutrient database), using Stata/IC 15.1(Stata, College Station, Texas, USA) (40).
- *Depression, Anxiety and Stress* will be assessed using The Depression, Anxiety and Stress Scale (DASS) (short-version) which has 21 items in 3 scales: depression (DASS-D-7 items), anxiety (DASS-A-7 items), and stress (DASS-S-7 items) (41). The items are scored on a 4-point Likert-type scale of 0 to 3 (0 = not at all, 3 = most of the time), and the total scores for each scale are to be multiplied by 2. The total score for each scale may range from 0 to 42, with higher scores indicating more depression, anxiety and stress. The internal consistency coefficient values (Cronbach's alpha) of each subscale ranges between 0.81 and 0.97 (41).
- *Quality of life* will be assessed using the Quality of Life Enjoyment and Satisfaction Questionnaire Short Form (Q-LES-Q-SF), which requires participants to report their satisfaction with their physical health, feelings, work, household duties, school/course work, leisure time activities and social relations (42). Scores range from 0 to 100 and higher scores indicate greater life satisfaction and enjoyment. The Q-LES-Q-SF internal consistency and retest reliability correlation coefficients were 0.90 and 0.93, respectively (43). The Satisfaction with Life Scale (SWLS) has 5 questions had requires participants to rate their response on a 7-point Likert scale from strongly disagree (1) to strongly agree (7), with higher scores reflecting greater satisfaction with life (44). The possible range of scores from this scale is 5–35. The SWLS has been shown to be a valid and reliable measure scale compared to other life satisfaction assessment measures (45).
- *Breastfeeding Practices:* Participants will be asked if they are currently breastfeeding, and if so they will be asked to indicate their child's date of birth they are breastfeeding, and whether the child consumes solid food, cow's milk or substitutes or infant formula. The responses will be used to determine whether the child is exclusively, complementarily, or not breastfed according to the WHO definitions (46).

### Other Measures

At baseline participants will be asked about their age, country of origin, language spoken at home, highest level of education, individual and household income, marital status, postcode, working status, and living situation/family structure. They will also be asked about their pregnancy history (number of pregnancies, their outcome and whether they were complicated by preeclampsia or other pregnancy complications) and their awareness of their increased risk of CVD at baseline and 3-months. Participants will be asked whether a health professional had provided advice or screening regarding CVD risk factors as per the SOMANZ Guidelines (9) since their most recent pregnancy with preeclampsia, to identify if any treatment was received during the trial.

### Statistical Methods

All analyses will be performed using Stata/IC (Stata, College Station, Texas, USA). Data will be presented as mean, standard deviation (SD) or median, interquartile range (IQR) for continuous variables and counts (percentages) for categorical variables. Changes in the impact on absolute CVD risk score, CVD risk markers and health behaviors will be determined, and differences between groups will be examined. Analysis for the preliminary efficacy outcomes will be conducted on an intention-to-treat basis (all participants who were randomized to groups and completed baseline assessments) and for completers only (those who provided data at 3-months). The effect of treatment on the efficacy outcomes will be assessed using linear mixed models. The efficacy outcome will be the outcome in the model, time (baseline, 3-months) and treatment group (intervention, control) as predictors, and group × time as an interaction term. The *p*-value of the interaction term will be used to determine the statistical significance of any difference between treatment groups in the change from baseline. Effect sizes will be calculated using the equation: Cohen's *d* = (M_1 change score_ – M_2 change score_)/SD_pooled (change scores)_. Intervention acceptability will presented as the mean ± SD, with higher scores (maximum of 5) indicating greater acceptability. For qualitative data analysis, answers from open questions will be categorized into themes.

## Discussion

Women with a history of preeclampsia have an elevated lifetime risk of premature cardiovascular related morbidity and mortality. While risk modification is recommended, there is currently limited evidence to guide the adoption and implementation of behavioral strategies to improve modifiable risk factors to promote cardiovascular health among this high-risk target group.

Strengths of this pilot RCT include the collection of data on the acceptability of a web-based behavioral intervention for women with a history of preeclampsia, as an important first step in the translation of health programs into clinical practice. Participant satisfaction and usage data will be used to determine whether the target population find the intervention acceptable, and whether any refinements are required prior to further testing. Additionally, this trial will provide an indication of the potential intervention effect on important markers of cardiovascular health and modifiable risk factors, through the evaluation of secondary outcomes. Evaluation of primary and secondary outcomes will also provide evidence of the feasibility of the data collection procedures for future trials with this target population and/or intervention. Finally, the study will provide important data to evaluate the potential reach and effectiveness of a variety of recruitment strategies to potentially identify the best setting(s) to reach women with a history of preeclampsia.

There are also some limitations to the study protocol to be acknowledged. Firstly, although the recruitment strategies are varied, and will give an indication of potential settings to reach women with a history of preeclampsia, they will recruit a convenience sample of women, and therefore introduce potential selection bias. Secondly, as this is a pilot RCT, the study is not powered to detect changes in outcomes. Thirdly, the follow-up period for assessment of preliminary efficacy of the intervention is immediately post-intervention. Therefore, the study will not evaluate changes to modifiable risk factors or markers of cardiovascular health beyond the 3-months time point. Finally, some preliminary efficacy outcomes may introduce measurement error, due to lack of sensitivity in the target group (e.g., Framingham CVD 30 year risk score), lower accuracy compared to other measurement devices (e.g., use of automated blood pressure monitor), or the self-reported nature of the measures (i.e., measurement of modifiable risk factors).

Overall, the current pilot RCT protocol comprehensively describes the methods to be used to evaluate the acceptability and preliminary efficacy of a web-based intervention developed specifically for women with a history of preeclampsia. If the pilot RCT demonstrates the acceptability and preliminary efficacy of the intervention approach, the next step will be to evaluate the efficacy of the intervention in a fully powered RCT, evaluating both post-intervention and longer-term impact on modifiable risk factors and markers of cardiovascular health. Findings of the pilot RCT will also guide the design of the RCT including recruitment strategies, data collection procedures, and sample size calculations, as well as inform any changes to intervention design. Study findings will also have broader applications to researchers and clinicians working with women with a history of preeclampsia, as they have potential to provide evidence of support for web-based interventions for risk factor modification, which may be used to inform the delivery of cardiovascular preventative health services.

## Author Contributions

MH conceptualized the research project. RT and MH drafted the manuscript. All authors were involved in the design of the study, edited and provided feedback, read, and approved the manuscript. The content in this manuscript is the original work of all authors involved.

### Conflict of Interest

The authors declare that the research was conducted in the absence of any commercial or financial relationships that could be construed as a potential conflict of interest.
